# Genomic characterization and pathogenicity validation of *Mycoplasma ovipneumoniae* strains from diseased sheep: insights into potential pathogenic mechanisms of GSWW364

**DOI:** 10.1186/s13567-026-01810-1

**Published:** 2026-07-07

**Authors:** Qiangsheng Lu, Jie Peng, Zongwu Ma, Lvfeng Yuan, Zhipeng Zhao, Shangdong Jia, Meijuan Tian, Huibin Tian, Xiaoxue Zhang, Qiaoying Zeng, Weimin Wang

**Affiliations:** 1 College of Veterinary Medicine, Gansu Agricultural University, Lanzhou, China; 2 State Key Laboratory of Herbage Improvement and Grassland Agro-Ecosystems; Key Laboratory of Grassland Livestock Industry Innovation, Ministry of Agriculture and Rural Affairs, Engineering Research Center of Grassland Industry, Ministry of Education, College of Pastoral Agriculture Science and Technology, Lanzhou University, Lanzhou, China; 3 Lanzhou Veterinary Research Institute, Chinese Academy of Agricultural Sciences, Lanzhou, China; 4 College of Animal Science and Technology, Gansu Agricultural University, Lanzhou, China

**Keywords:** *Mycoplasma ovipneumoniae*, genomic characterization, Hu sheep, host pathogenesis, Mo GSWW364

## Abstract

*Mycoplasma ovipneumoniae* (Mo) is the primary etiological agent of mycoplasmal pneumonia of sheep in China, posing a significant threat to the sheep industry. In this study, 18 strains of Mo were isolated from nasal swab samples collected from diseased Hu sheep at nine farms in Gansu Province. Nine representative strains were selected for whole-genome sequencing (WGS) and analysis to explore the genetic relationships between the isolates and reference strains from different regions and hosts. On the basis of the cultural characteristics and the genetic variability among regional strains, strain GSWW364 was chosen for further experiments. A total of 100 healthy 2-month-old Hu sheep were challenged experimentally with strain GSWW364 at a dose of 1 × 10^7^ color change units (CCU). Fifty-four sheep exhibited typical clinical symptoms, including elevated body temperature, coughing, and pulmonary lesions, confirming the pathogenicity of GSWW364. Transcriptomic analyses at both the tissue and cellular levels revealed three infection-associated differentially expressed genes (DEGs): *HIF1A*, *SLC2A3*, and *SLC38A1*. Validation confirmed significantly upregulated expression of these genes in diseased lung tissues. Knockdown of these genes in sheep alveolar epithelial type II cells significantly reduced Mo adhesion. These findings suggest that these genes may promote pathogen adhesion and inflammatory responses by regulating hypoxia-responsive and metabolic pathways during infection. Collectively, this study provides a scientific basis for the prevention and control of mycoplasmal pneumonia of sheep and lays the groundwork for further investigation into the disease’s pathological mechanisms. It also offers valuable insights for vaccine development and disease-resistant breeding programs.

**Graphical Abstract:**

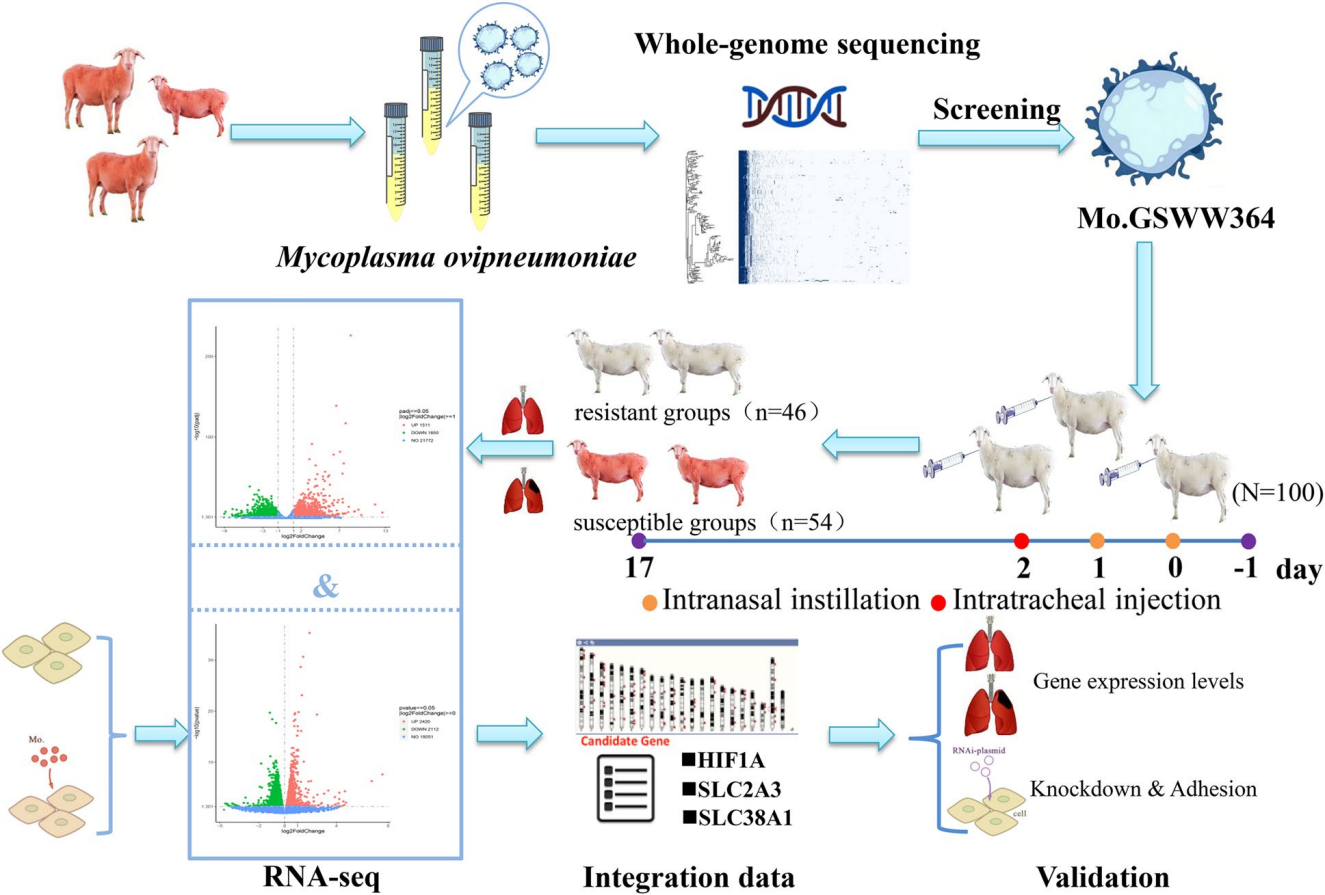

**Supplementary Information:**

The online version contains supplementary material available at 10.1186/s13567-026-01810-1.

## Introduction

Mycoplasmal pneumonia of sheep is a chronic respiratory disease characterized by interstitial and proliferative lesions and nonspecific clinical signs, including coughing, dyspnea, fever, and weight loss [1, 2]. The disease is widely distributed and is associated with impaired host immunity, increased susceptibility to secondary infections, and substantial economic losses in sheep production systems [3–5]. Mo is the principal etiological agent [6] and has been reported in multiple regions of China [7–10].

Understanding the genetic characteristics of Mo is essential for elucidating its pathogenicity and epidemiology. Conventional identification and phenotypic methods provide limited resolution [11], while molecular typing approaches such as pulsed-field gel electrophoresis (PFGE) and multilocus sequence typing (MLST), although informative, are constrained by partial genome coverage and technical complexity [12, 13]. WGS enables comprehensive analysis of genetic diversity and evolutionary relationships and has become an effective tool for Mo research [14].

Host factors are critical determinants of disease outcome. Variation in susceptibility to Mo infection has been observed both between breeds and among individuals within the same breed, with lambs showing increased susceptibility [15, 16]. Hu sheep, an important commercial breed characterized by high productivity and early maturity, are widely used in mutton production systems [17]. Field observations indicate substantial economic losses associated with mycoplasmal pneumonia in this breed [6], together with marked interindividual variation in susceptibility [6], suggesting a role for host genetic determinants. In this study, healthy Hu sheep were experimentally infected with Mo and classified into susceptible and resistant groups on the basis of clinical phenotypes to facilitate the analysis of host responses.

RNA sequencing (RNA-Seq) is a powerful approach for identifying genes involved in host-pathogen interactions [18], but most studies have focused on single tissues and lack integrated in vivo and in vitro analyses [19, 20]. Here, RNA-seq was performed on lung tissues and alveolar epithelial cells, and the datasets were integrated to identify DEGs associated with disease.

In this study, we combined Mo isolation, WGS, and multilevel transcriptomic analyses to characterize pathogen diversity and host responses. These findings provide insights into the pathogenesis of mycoplasmal pneumonia and a basis for improving disease resistance in Hu sheep.

## Materials and methods

### Strains and cells

#### Isolation, culture, and identification of pathogens

The Mo strain Y98 was purchased from the American Type Culture Collection (ATCC, Manassas, VA, USA) and preserved in our laboratory.

Complete medium (1 L) was prepared by dissolving 21 g of pleuropneumonia-like organisms (PPLO) broth powder (255,420, BD, USA) and 3 mL of 1% (w/v) Phenol Red in distilled water and adjusting the volume to 600 mL. The pH was titrated to 7.8-8.0 with 1 mol/L NaOH, and the solution was sterilized at 121 °C for 20 min. After cooling to room temperature, 100 mL of 20% (w/v) yeast extract, 200 mL of horse serum (ZPRM200-08, Zhengzhou Pingrui Biotechnology Co., Ltd., China), 100 mL of minimum essential medium (MEM; 11,095-080, Gibco, USA), and 1 mL of 50 mg/mL ampicillin sodium salt were added aseptically. The medium was subsequently stored at 4 °C for short‑term use.

Solid medium was prepared by adding an additional 1.5% agar to the above formulation.

Nasal swabs were collected from 21 sheep showing obvious clinical symptoms of pneumonia at 9 sheep fattening farms in Gansu Province, all of which had clinical cases of mycoplasmal pneumonia. The samples were obtained from sheep aged 3-5 months, sourced from breeding farms across Jiangsu, Shandong, Zhejiang, Gansu, and Tianjin. The swabs were stored in sterile test tubes containing complete medium, appropriately labeled, and sent to the laboratory for pathogen isolation and identification.

Under sterile conditions, 1 mL of each swab-medium mixture was inoculated into 5 mL of fresh medium and incubated statically at 37 °C for 48 h. After filtration through a 0.22-μm membrane, the medium changed color from red to yellow, indicating bacterial growth. Subcultures were then performed at a 1:10 ratio (bacterial suspension to fresh medium). Once the medium consistently turned yellow without visible sediment within 48 h, the bacterial suspension was aspirated and spread onto solid agar medium at 5% CO_2_ and 37 °C for 5-7 days. Single colonies were picked and transferred to liquid medium for amplification. The purified strains were stored at -80 °C for future use.

Genomic DNA was extracted from the bacterial suspension using a DNA extraction kit (Shanghai Glinx Biotechnology Co., Ltd., China), and the extracted DNA was used as a template for PCR amplification (P223-01, Vazyme, China). The amplification was performed using the primer pair Mo-16 s-F (5′-GACTTCATCCTGCACTCTGT-3′) and Mo-16 s-R (5′-TGAACGGAATATGTTAGCTT-3′). The PCR reaction system and program are shown in Additional files 1, 2. The PCR products were analyzed by 1% agarose gel electrophoresis, and the 361-bp target bands were sent for sequencing. After 48 h of cultivation, bacteria corresponding to 10^8^ CCU were harvested and resuspended in 2.5% glutaraldehyde for fixation. The samples were then sent to Sichuan Lilaisinuo Biotechnology Co., Ltd. for scanning electron microscopy (SEM) to observe bacterial morphology.

#### Whole-genome sequencing and analysis of isolated strains

Nine Mo strains isolated from different sheep farms (designated as GSWW364, GSWW590, GSWW170, GSWW195, GSWW349, GSWW350, GSWW420, GSWW462, and GSWW463) were selected, genomic DNA was extracted from the cultured isolates. All other 168 Mo strains sequences were downloaded from the National Center for Biotechnology Information (NCBI) database, except for those isolated in this study. The relevant information for the other 168 Mo strains is provided in Additional file 3. WGS was performed on the Illumina NovaSeq platform using paired-end 150 bp (PE150) libraries, yielding ≥ 5 Gb of raw data. The raw sequencing data were filtered using Trim Galore (version 0.6.10, RRID: SCR_011847), followed by microbiome analysis using PathSeq (GATK version 4.6.0.0, RRID: SCR_001876). The filtered sequences were aligned to the Mo reference genome Y98 (GCF_028885435) using BWA (version 0.7.15, RRID: SCR_010910). Alignment statistics were quantified and summarized using BEDtools (version 2.31.1, RRID: SCR_006646). In addition to the nine Mo strains in this study, the genome sequences of all other Mo strains were downloaded from public databases and merged with the sequencing data generated in this study. Genomic variant calling was performed using GATK, generating variant call format (VCF) files, which were filtered using VCFtools (version 0.1.16, RRID: SCR_001235) with the following criteria: 2 alleles, a missing individual rate ≤ 0.1, and a minor allele frequency (MAF) ≥ 0.02. Missing values were imputed with BEAGLE (version 5.5, RRID: SCR_001789) to generate high-quality VCF files.

After concatenating the whole-genome variant sites, a phylogenetic tree was constructed using MEGA11 (RRID: SCR_000667). Principal component analysis (PCA) was performed on the high-quality VCF files using PLINK2 (version 2.00a, RRID: SCR_001757). Cluster analysis and *t*-distributed stochastic neighbor embedding (*t*-SNE) dimensionality reduction were performed using the scikit-learn (sklearn) (RRID: SCR_002577) package in Python 3. Genome sequencing data were assembled using Shovill (version 1.1.0, RRID: SCR_017077), and the genomic sequence fragments of GSWW364 were reordered against the Y98 genome using MAUVE (version 20150226, RRID: SCR_012852) for genomic collinearity analysis. The genomic sequence of GSWW364 was annotated using Prokka (version 1.14.6, RRID: SCR_014732).

### Susceptibility and pathology of sheep infected with Mo GSWW364 strain

A total of 110 healthy, 2-month-old weaned Hu sheep, with equal numbers of males and females, were purchased from Shandong Linqing Runlin Animal Husbandry Co., Ltd. (China). These sheep had not been immunized with the Mo vaccine. All sheep were housed at the Joint Experimental Base of Lanzhou University and Gansu Agricultural University for subsequent investigations. Prior to the experiment, the entire facility underwent thorough disinfection. Upon arrival, the sheep were acclimatized for 7 days to alleviate transportation and environmental stress, and the experiments were initiated only after ensuring the animals’ physiological and behavioral stability.

The experimental group consisted of 100 sheep, which were infected with Mo. For three consecutive days, they received two intranasal instillations and one intratracheal injection, each with a dose of 1 × 10^7^ CCU/mL. Additionally, 10 age- and weight-matched sheep formed the sham infection group (SIG), which was inoculated with sterile culture medium using the same inoculation method, in order to control for other variables, such as the effects of the handling procedures.

During the experiment, rectal temperature was measured at fixed times daily. From the third day post-infection until necropsy, the cough frequency was observed for 1 h each in the morning and afternoon, with observations scheduled outside feeding times. Blood samples were collected on the day prior to inoculation and every 3 days thereafter. Mo antibodies were detected using indirect enzyme-linked immunosorbent assay (ELISA), with S/P ≥ 0.20 considered positive. On the basis of body temperature, cough frequency, and antibody levels, sheep were preliminarily classified as susceptible or resistant: continuous fever (body temperature ≥ 40 °C for ≥ 3 days), severe coughing (frequency > 0.20 times/h), and sustained elevation in serum antibody levels were criteria for positivity.

Two weeks after the infection period, body weights were measured. All sheep were euthanized by electrical stunning followed by exsanguination (GB/T 39760-2021 and GB/T 42071-2022), and necropsy and weighing were performed. Disease was further confirmed through post mortem lung lesions, bacterial load in lung tissue, and the size of the hilar lymph nodes. Lung tissues were immediately fixed in 4% paraformaldehyde and sent for company-based sectioning, hematoxylin and eosin (H&E) staining, and microscopic examination. DNA extraction was also performed, and Mo-specific Php gene (primers: Mo-Php-F 5′-GCCCTGAAGCTCATGAACACC-3′, Mo-Php-R 5′-ATCAATTGCCCCATAACCTGT-3′) was quantified via quantitative polymerase chain reaction (qPCR; Q711-02, Vazyme, China) to assess bacterial load in the lung tissue. The qPCR reaction system and program are shown in Additional files 4, 5.

### Transcriptomic sequencing and analysis at the tissue and cellular levels

Sheep alveolar epithelial type II (SAT II) cells were isolated and obtained from the Laboratory of Microbiology and Immunology, Gansu Agricultural University. Lung samples from three clinically affected sheep and three healthy controls from the experimental group were snap-frozen in liquid nitrogen, while SAT II cells were infected with strain GSWW364 at a multiplicity of infection (MOI) of 100 for 2 h (with uninfected cells serving as negative controls); all samples were collected and sent for transcriptomic sequencing to Beijing Novogene Bioinformatics Technology Co., Ltd. (China). Both tissue-level and cell-level transcriptomic analyses were performed using bulk RNA-seq. DEGs at the tissue level were screened using thresholds of |log_2_ FC|> 1 (where FC is the fold change) and *P*_adj_ < 0.05. Given the higher heterogeneity of tissue samples, this stringent threshold was applied to control false positives and ensure reliable results. In contrast, for the cellular-level transcriptome, DEG screening was performed using the following criteria: |log_2_ FC|> 0 and *P* < 0.05. The relatively homogeneous background of in vitro cell models allowed a relaxed threshold to comprehensively capture potential DEGs without excessive filtering. Subsequently, Gene Ontology (GO) functional enrichment analysis and Kyoto Encyclopedia of Genes and Genomes (KEGG) enrichment analysis was conducted to identify the major biochemical metabolic and signal transduction pathways involved in these DEGs. Ultimately, an integrated analysis was performed based on the transcriptomic datasets obtained from the tissue and cellular levels.

### Screening and validation of pathogenic genes associated with mycoplasmal pneumonia of sheep

On the basis of the transcriptomic sequencing results, three DEGs—*HIF1A*, *SLC2A3*, and *SLC38A1*—were selected for further analysis. The three key genes were identified as the overlapping DEGs shared between the tissue-level and cellular-level transcriptome analyses, and we selected the most significantly differentially expressed and commonly upregulated genes from the transcriptomes for further validation. The β-tubulin gene (*TUBB*) was used as the reference gene, and specific primer pairs for qPCR were designed (Table 1). qPCR assays were then performed to compare the expression levels of these three genes in the lung tissues from both susceptible and resistant groups (*n* = 100).

**Table 1:** **The primer sequences of real-time quantitative PCR**

	**Primer**	**Primer sequence**
*HIF1A*	*HIF1A*-qF	TTCCAGCCCACTCAAATGCAA
	*HIF1A*-qR	TTTCCTGCTCTGTTGGGTGA
*SLC2A3*	*SLC2A3*-qF	TCTCGATTGCTACCATAGGCTC
	*SLC2A3*-qR	CCAATCATACCACCCACGGA
*SLC38A1*	*SLC38A1*-qF	CAGTCATTGTGGCCGTCA
	*SLC38A1*-qR	CGCTGAGTTCCCTTATCCC
*TUBB*	*TUBB*-qF	ACAGGTGGCAAATATGTCCCT
	*TUBB*-qR	GGCCTCCTTCCGAACCACA

Knockdown of these three genes was performed in SAT II cells. The siRNAs targeting these genes were purchased from Shanghai Jiman Biotechnology Co., Ltd. (China) with sequence information provided in Table 2. Transfection was performed using the Lipofectamine^®^ 3000 Kit (L3000-015, Thermo Fisher Scientific, USA). Forty-eight hours post-transfection, the siRNA-transfected cells were infected with strain GSWW364 at an MOI of 100 for 2 h, and the adhesion rate was detected to compare with the control group and investigate whether the knockdown of these three genes affects the adhesion rate of strain GSWW364.

**Table 2:** **siRNA sequence information**

siRNA name	siRNA sequence
*HIF1A*(s)-si	CAGCUAUUUGCGUGUGAGGAATT
	UUCCUCACACGCAAAUAGCUGTT
*SLC2A3*(s)-si	CUACUCGACAGGAAUCUUCAATT
	UUGAAGAUUCCUGUCGAGUAGTT
*SLC38A1*(s)-si	CGUUGGCGUUACAUCUGCUAATT
	UUAGCAGAUGUAACGCCAACGTT
siRNA NC	UUCUCCGAACGUGUCACGUdTdT
	ACGUGACACGUUCGGAGAAdTdT

### Statistical analysis

Biological and technical replicates were analyzed using two-tailed unpaired Student’s *t*-test with GraphPad Prism 8.4.2 software (RRID: SCR_002798), and data are presented as the mean ± standard deviation (SD). Statistical significance is denoted as follows: ^ns^*p* ≥ 0.05; *p < 0.05; **p < 0.01; and ****p < 0.0001.

## Results

### Isolation and identification of clinically isolated strains

**Figure 1:**
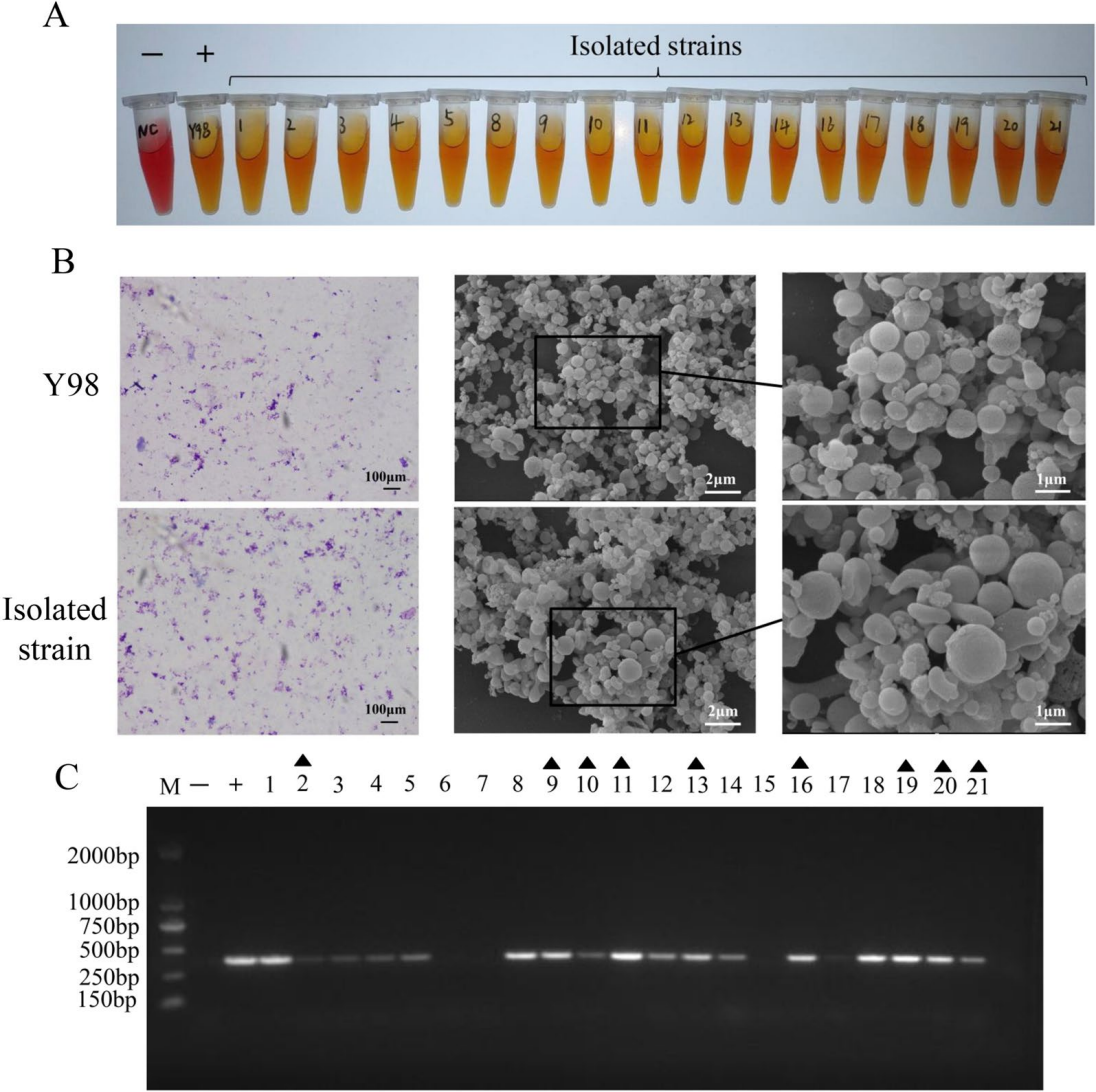
**Identification results of the strains.** **A** Cultural characteristics of the isolated strains (“–”: negative control; “ + ”: positive control strain Y98; “Isolated strains”: strains isolated from the farms). **B** Morphological observation of the strains. Column 1: Giemsa staining results (100 × magnification); Columns 2 and 3: SEM results (magnification: 8,000× for column 2; 20,000× for column 3). (“Y98”: standard strain; “Isolated strain”: representative isolated strain). **C** PCR amplification results of the 16S rRNA gene. (“M”: DL2000 DNA Marker (TaKaRa, Japan, cat. no. 3427 A); “–”: negative control; “ + ”: positive control strain; “1–21”: presumptive strains isolated from the farms; “▲”: selected strains from nine sheep farms in Gansu Province).

A total of 18 suspected Mo strains were isolated from diseased sheep with mycoplasmal pneumonia from different farms in Gansu Province. These strains proliferated in liquid medium with Phenol Red, causing the medium to change color from red to yellow owing to acid production (Figure 1A). Giemsa staining revealed bluish-purple bacterial cells (Figure 1B), and SEM displayed distinct pleomorphism (Figure 1B). The morphological and staining characteristics of the isolated strain were consistent with those of the Mo Y98 reference strain.

To further identify these strains, we performed PCR amplification of the 16S rRNA gene using specific primers targeting the Mo. The amplification results showed that all isolates produced a consistent 361-bp target band (Figure 1C). The amplified products were sequenced, and the sequencing results were submitted to the NCBI database for homology comparison, confirming that all isolates were Mo.

### Whole-genome sequencing and analysis of isolated strains

**Figure 2:**
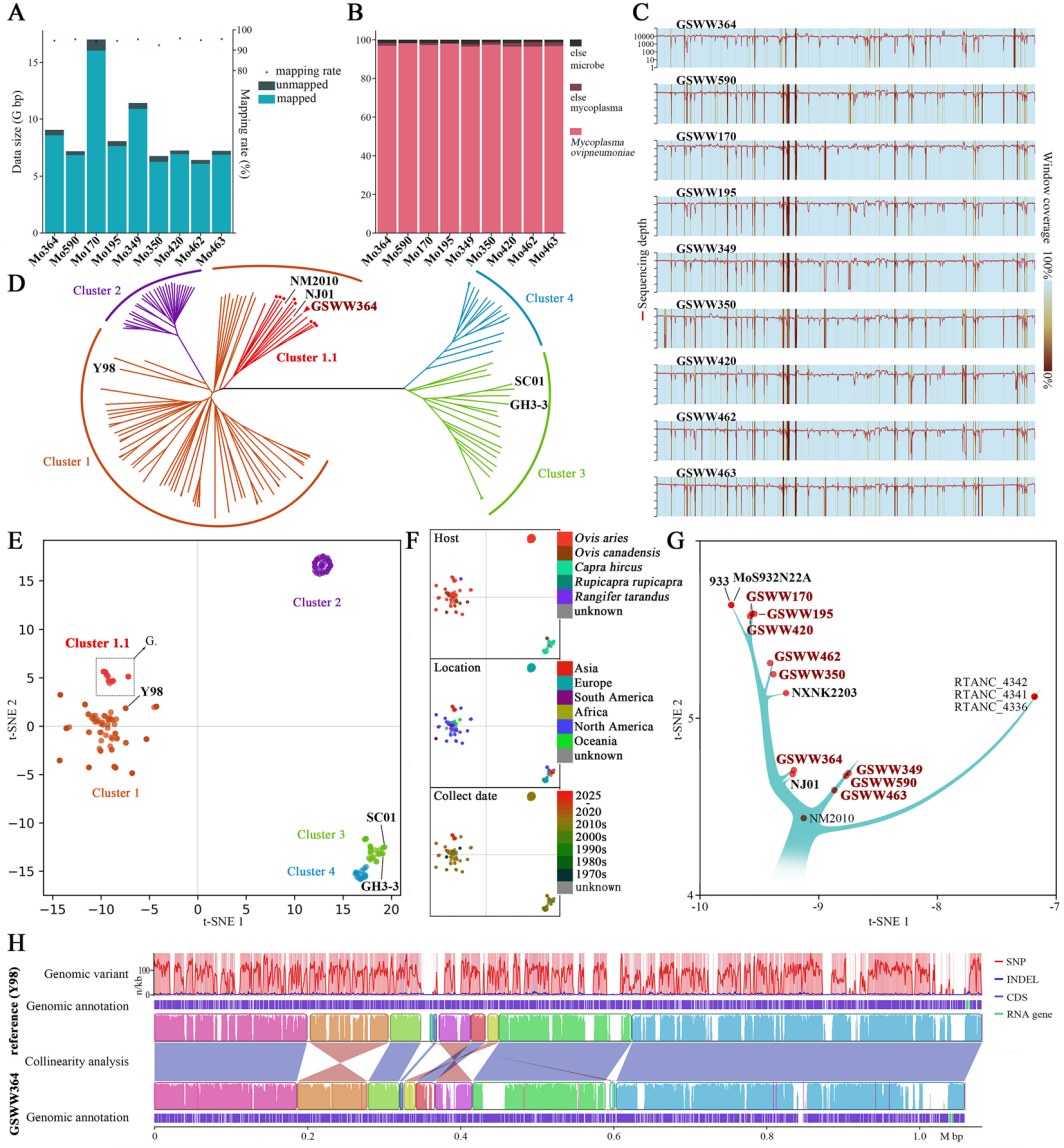
**Genomic analysis of Mo isolates.** Nine representative strain labels follow “Mo” (**A**, **B**) or “GSWW” (**C**, **D**, and **G**) with identical numerical suffixes indicating the same isolates (e.g., Mo364 = GSWW364). **A** Plot of genomic sequencing data volume. The left *y*-axis represents the sequencing data volume, and the light-blue bars indicate the data volume of sequences successfully mapped to the Mo reference genome (Y98, GCF_028885435). The dark-blue bars represent other sequences. The right *y*-axis denotes the Mo mapping rate, which is indicated by dots. **B** Microbiome analysis. Red indicates the proportion of Mo-related sequences, dark red denotes the proportion of sequences related to other mycoplasmas, and black represents the proportion of sequences related to other microorganisms. **C** Plot of genomic sequencing coverage. Sliding window analysis of read depth across the reference genome (Y98), with heatmap indicating coverage breadth. **D** Phylogenetic tree. Phylogenetic tree constructed on the basis of whole-genome variant locus information. Major clades were distinguished by different colors. Nine representative strains isolated and sequenced in this study are marked with red dots and red triangles, and important strains are labeled with text annotations. **E** Scatter plot of principal component dimensionality reduction based on whole-genome variations. Important strains are labeled with text annotations. The magnified range in **G** is indicated by a dashed box. **F** Plot of strain information annotation. From top to bottom, the plots represent the host species, sampling location, and collect date, respectively. **G** Magnified view of **E**. Strains isolated and sequenced in this study are indicated with red text. Other closely related strains are labeled with black text: relevant information for these strains are provided in the Supplementary Material. **H** Plot of genomic collinearity analysis. From top to bottom, the plots are as follows: plot of genomic variant locus distribution, gene annotation plot of the Y98 genome, collinearity analysis plot between the Y98 and GSWW364 genomes, and gene annotation plot of the GSWW364 genome.

The sequencing results showed that the 16S rRNA gene sequences of Mo strains isolated from the same farm were consistent. Therefore, we selected nine Mo strains from different farms for WGS, generating over 6 Gbp of data. The alignment rate with the Mo reference genome Y98 was approximately 95% (Figure 2A). Microbiome analysis indicated low contamination risk (Figure 2B). The sequencing depth reached approximately 10^4^, with 100% coverage (Figure 2C).

On the basis of the WGS of these nine strains, together with data from 126 Mo strains from the sheep-associated lineage and 42 Mo strains from the goat-associated lineage retrieved from the NCBI database, a phylogenetic tree was constructed. This revealed that isolates from sheep clustered in clusters 1 and 2, while isolates from goats were in clusters 3 and 4 (Figure 2D). These results suggest significant differences between Mo strains from different host species. Variations were also observed among Mo isolates derived from sheep. Our strain clustered with other Chinese sheep-derived strains in the evolutionary branch cluster 1.1.

PCA was used for dimensionality reduction of genomic variation data, revealing overall genetic clustering and population structure across the samples. *K*-means clustering divided the strains into four distinct clusters, named cluster 1 to cluster 4, on the basis of the reduced genomic variation data, identifying potential population differentiation or genetic lineages. Subsequently, *t*-SNE analysis was performed on the first 19 principal components to visually validate the *K*-means clustering results. This analysis displayed the distribution patterns and similarities of strains within and between clusters (Figure 2E). A scatter plot annotated with strain information, including host species, sampling location, and collection date, is provided in Figure 2F.

Clusters 1 and 2 consisted of Mo strains from sheep. Isolates from Iceland formed a separate cluster (cluster 2) owing to their larger number and smaller variation. Mo strains from China clustered with some strains from Austria and the USA in cluster 1.1. Additionally, Mo strains from goats were further divided into two subgroups: the European cluster (cluster 4) and other regions (cluster 3). Notably, strains such as GH3-3 and SC01, although isolated from China, were genetically distant from sheep-derived isolates due to their goat host origin. The *t*-SNE plot focused on the region corresponding to cluster 1.1, with a phylogenetic tree based on the phylogenetic data of the strains. The phylogenetic tree and PCA scatter plot based on whole-genome variation showed significant genetic differentiation among the Mo strains in clusters 1-4. This indicates substantial differences between isolates from different hosts and geographic regions. Phylogenetic analysis of cluster 1.1 further revealed genetic variation among the nine Mo strains in this study. In the magnified view of Figure 2E, strain GSWW364 showed a close genetic relationship to the highly pathogenic Mo strain NJ01 (Figure 2G). Thus, we hypothesize that GSWW364 is a highly virulent Mo strain. To further investigate the genomic evolution of GSWW364, we performed collinearity analysis between the genome of GSWW364 and the international reference strain Mo Y98. This revealed differences in genetic loci, local sequences, and genomic structures (Figure 2H). Collinearity analysis between GSWW364 and the strain NJ01, and between Y98 and NJ01, are provided in the Additional file 6. These results suggest that the GSWW364 strain holds significant genetic relevance and warrants further investigation into its pathogenicity.

### Susceptibility and pathology of sheep infected with Mo GSWW364 strain

In the preliminary investigation, significant individual differences in susceptibility to Mo infection were observed within the Hu sheep population. On this basis, a cohort of 100, 2-month-old Hu sheep was selected for an experimental infection study. This experiment aimed not only to evaluate the virulence of the Mo GSWW364 strain, but also to explore potential pathogenic mechanisms by leveraging the variability in susceptibility among individuals of the same breed.

**Figure 3:**
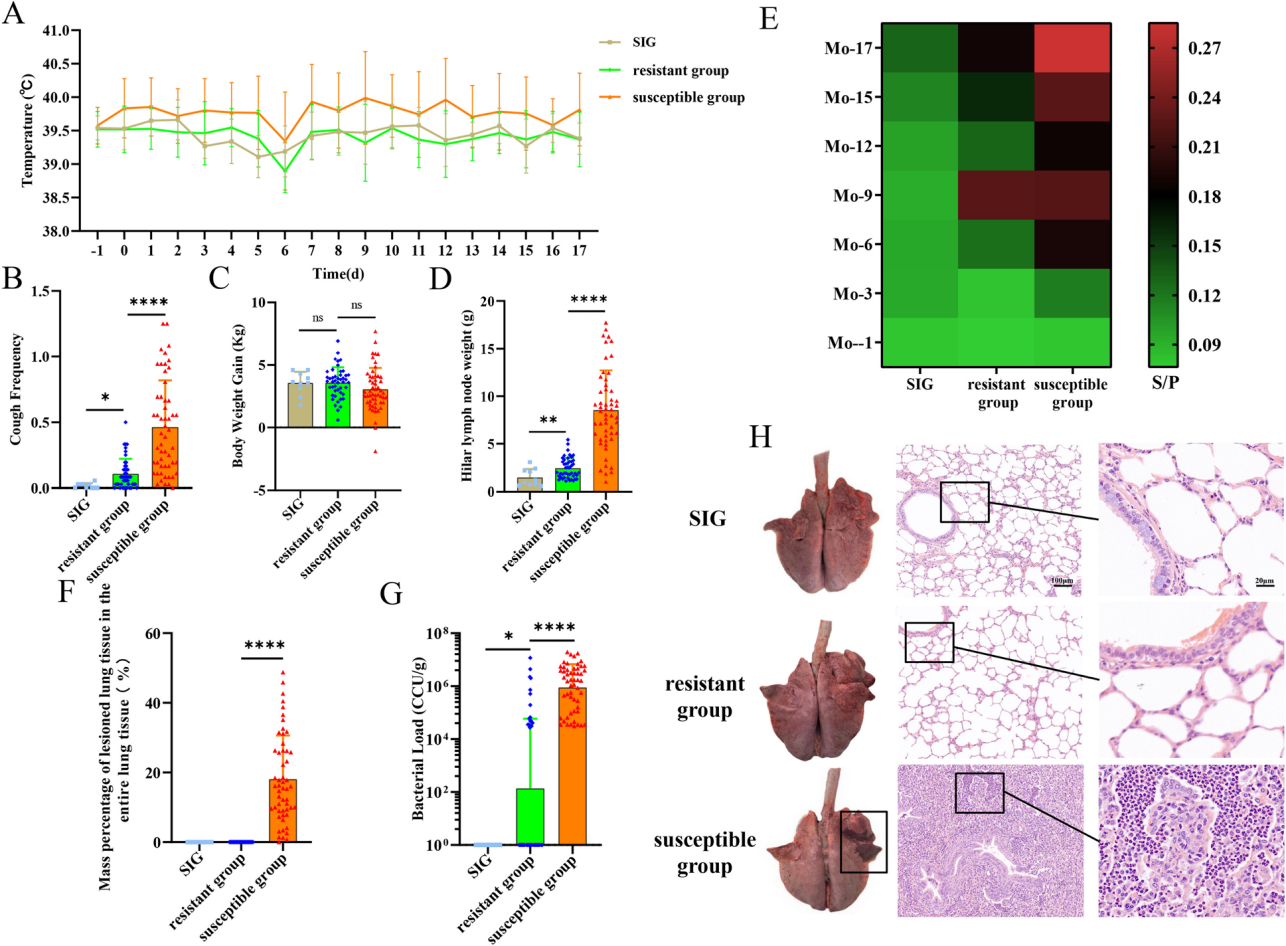
**Clinical indicators related to mycoplasmal pneumonia of sheep.** **A** Changes in body temperature. **B** Average cough frequency per hour. **C** Changes in sheep body weight gain before and after challenge inoculation. **D** Lymph node weight; **E** Changes in Mo antibody levels. (“Mo” refers to the challenge strain GSWW364, and the notation “Mo-number” denotes the corresponding days post-challenge. Challenge was administered consecutively for 3 days on days 0, 1, and 2. “Mo--1” indicates the day before challenge; “Mo-3” indicates the first day post-challenge; “Mo-6” indicates the fourth day post-challenge; “Mo-9” indicates the seventh day post-challenge; “Mo-12” indicates the tenth day post-challenge; “Mo-15” indicates the thirteen day post-challenge; “Mo-17” indicates the fifteenth day post-challenge.). **F** Mass ratio of lesioned lung tissue to total lung tissue. **G** bacterial load. **H** observation of pathological changes in lung tissues. The first column shows the gross observation results of lung tissues; the second and third columns show the H&E staining results of lung tissue sections (magnification: 10× for the second column; 40× for the third column.). (“SIG” indicates sheep in the sham infection group, “resistant group” indicates non-diseased sheep in the experimental group, and “susceptible group” indicates diseased sheep in the experimental group.). (In the bar charts of **B**, **C**, **D**, **F**, and **G**, the groups from left to right are the SIG group, the resistant group, and the susceptible group, respectively).

Following infection, 54 sheep developed typical clinical signs of mycoplasmal pneumonia (susceptible group), whereas 46 sheep showed no obvious clinical symptoms (resistant group). The results showed that body temperature in the susceptible group increased significantly after inoculation, while it remained within the normal range in both the resistant group and the SIG group (Figure 3A). The coughing frequency in the susceptible group was markedly higher than that in the other two groups (Figure 3B), and body weight gain was slower (Figure 3C). Necropsy findings revealed typical hepatization in the lungs of the susceptible group (Figure 3H), with a significantly higher proportion of lesion areas (Figure 3F), along with enlarged lymph nodes (Figure 3D). In addition, the bacterial load in lung tissues of the susceptible group was significantly higher than that in the resistant group (Figure 3G). Serological analysis by ELISA showed that Mo-specific antibody levels in the susceptible group continuously increased, whereas in the resistant group, antibody levels peaked and then gradually returned to normal levels (Figure 3E). Histopathological examination (H&E staining) demonstrated severe destruction of alveolar structures accompanied by extensive inflammatory cell infiltration in the susceptible group. In contrast, the alveolar structures in both the resistant group and the SIG group remained intact and morphologically normal (Figure 3H).

Therefore, Mo GSWW364 is a pathogenic strain with clear virulence; however, its pathogenic effects vary significantly among individual hosts. This variability is likely associated with host-specific differences. Therefore, further analysis of host responses to Mo infection across individuals may provide important insights into the underlying pathogenic mechanisms.

### Transcriptomic sequencing analysis of sheep in response to mycoplasmal pneumonia

**Figure 4:**
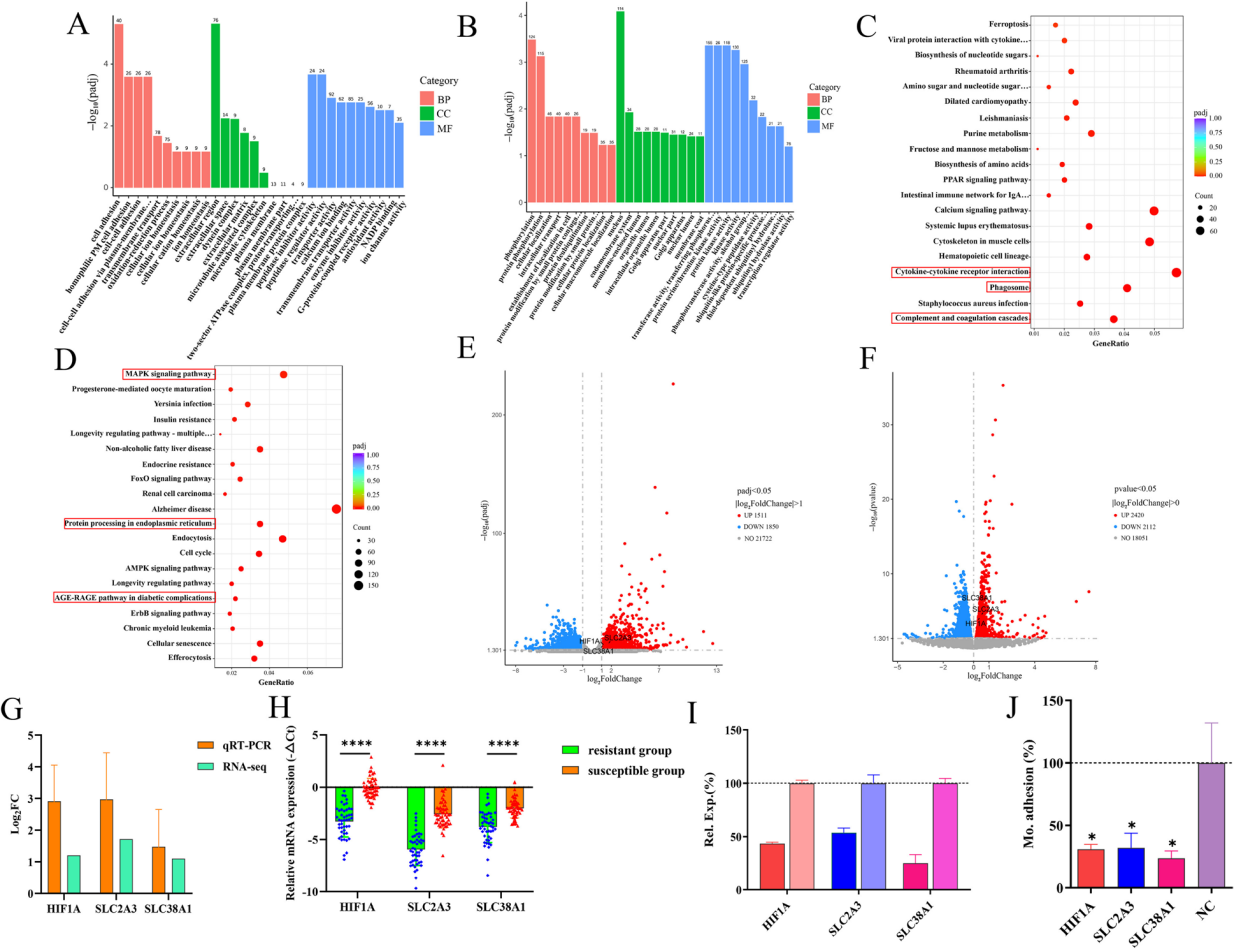
**Transcriptomic sequencing results and validation of DEGs.** **A** Histogram of the results of the GO functional enrichment analysis of DEGs in sheep lung tissues infected with Mo. **B** Histogram of the results of the GO functional enrichment analysis of DEGs in SAT II cells infected with Mo. BP, biological process; CC, cellular component; MF, molecular function. **C** Bubble chart of KEGG pathway enrichment analysis for DEGs in sheep lung tissues infected with Mo. **D** Bubble chart of KEGG pathway enrichment analysis for DEGs in SAT II cells infected with Mo. **E** Volcano plot of DEGs in transcriptome of sheep lung tissues infected with Mo. **F** Volcano plot of DEGs in transcriptome of SAT II cells infected with Mo. **G** Expression validation of transcriptome-screened genes in lung tissues from both susceptible and resistant groups (*n* = 100 total); FC, fold change in relative gene expression. **H** Relative mRNA expression levels of transcriptome-screened key genes in lung tissues from both susceptible and resistant groups (*n* = 100 total); The *y*-axis represents the −ΔCt value. The larger the −ΔCt value, the higher the gene expression level. **I** Determination of knockdown efficiency of genes identified by transcriptomic screening; The *y*-axis represents percentage relative expression of the corresponding gene in cells after gene knockdown. For each gene on the x-axis, the right bar indicates gene expression before knockdown, and the left bar indicates expression after knockdown. **J** Changes in Mo adhesion rate following knockdown of identified genes. For each gene on the *x*-axis, the corresponding bar represents the change in the adhesion rate of Mo GSWW364 to SAT-II cells after gene knockdown.

The heterogeneity of clinical infection phenotypes may arise from individual differences in host-pathogen interactions. As the lung is the primary target organ of Mo infection, this study first focused on the local host response characteristics in the lung, and identified host molecular response patterns associated with disease phenotypes via transcriptome analysis. Three lung tissue samples from each of the susceptible and resistant groups were selected for transcriptomic sequencing. The ten sheep of the SIG were not part of the main experimental group of 100 and were excluded from the final “susceptible” versus “resistant” analysis. The results of GO functional enrichment analysis demonstrated that, at the tissue level, Mo infection in lung tissues triggers pathogenic infection responses via cell-cell interactions, extracellular microenvironment modulation, and membrane transport (Figure 4A). Through KEGG enrichment analysis, it was found that Mo infection significantly activated inflammation- and immunity-related signaling pathways at the tissue level, including cytokine-cytokine receptor interaction, phagosome, and complement and coagulation cascades (Figure 4C).

SAT II cells represent the primary cellular targets of Mo infection. To dissect cell-intrinsic mechanisms underlying host pathogen interactions within a simplified system, this study further established an in vitro infection model of Mo in SAT II cells, aiming to identify epithelial-specific early response signatures through cell-level transcriptome analysis, thereby complementing the multicellular complexity inherent in tissue-level data. Three cell samples from Mo-infected and uninfected SAT II cells were selected for transcriptomic sequencing. The results of GO functional enrichment analysis demonstrated that, at the cellular level, Mo infection induces intracellular molecular modifications, organelle functional activation, and the initiation of relevant transcriptional regulatory programs (Figure 4B). Through KEGG enrichment analysis, it was found that, at the cellular level, in addition to inflammatory signaling pathways such as the mitogen-activated protein kinase (MAPK) and advanced glycation end-products and their receptor (AGE-RAGE) signaling pathways, significant enrichment was also detected in pathways including protein processing in endoplasmic reticulum and other key pathways (Figure 4D).

In conclusion, Mo infection can trigger inflammatory responses at both the tissue and cellular levels simultaneously. At the tissue level, it involves multicellular networks and the microenvironment. At the cellular level, it focuses on intracellular regulation. Together, these levels reveal the overall host response mechanism induced by Mo infection.

### Transcriptomic identification and validation of key genes associated with Mo infection

We further analyzed the transcriptomic sequencing results from both groups. Volcano plot analysis revealed a total of 3361 DEGs in the tissue-level transcriptome between the susceptible and resistant groups, with 1511 genes upregulated and 1850 genes downregulated (Figure 4E). At the cellular level, 4532 DEGs were identified between the infected and uninfected groups, with 2420 genes upregulated and 2112 genes downregulated (Figure 4F). A total of 187 common DEGs were identified between the two transcriptomic datasets, with 60 genes upregulated and 127 genes downregulated. Given that upregulated genes represent the host’s active response to pathogen invasion or pathological activation, we selected the most significantly differentially expressed common upregulated genes for further validation. Using the uniform genetic background of the in vitro cell model, we ensured that the selected targets exert distinct regulatory effects, facilitating subsequent mechanistic dissection. Ultimately, we chose hypoxia-inducible factor 1 alpha (*HIF1A*), solute carrier family 2 member 3 (*SLC2A3*), and solute carrier family 38 member 1 (*SLC38A1*) for validation (Figure 4E, F).

We first validated the expression levels of these three target genes in lung tissues from sheep with different clinical manifestations of Mo infection. The results were consistent with the transcriptomic data (Figure 4G), showing that the transcriptional levels of these three genes were significantly higher in the lung tissues of susceptible sheep compared with the resistant group (Figure 4H), further confirming the selection of these genes for subsequent study. We then performed preliminary validation of the roles of these three genes in Mo infection. Knockdown of *HIF1A*, *SLC2A3*, and *SLC38A1* in SAT II cells reduced their transcriptional levels (Figure 4I). Further Mo adhesion assays confirmed that, when the transcriptional levels of these genes were decreased, the adhesion rate of Mo to SAT II cells was significantly reduced (Figure 4J). These findings indicate that these three genes are indeed closely associated with Mo infection, but their precise mechanisms of action warrant further investigation.

## Discussion

Mycoplasmal pneumonia of sheep represents a major infectious disease responsible for substantial economic losses in the global sheep industry [21, 22]. Epidemiological studies indicate that the incidence of this disease ranges from 5% to 80% in China and from 20% to 88% worldwide [4, 10], underscoring its widespread occurrence in intensive sheep farming systems and its significant impact on animal health and production efficiency.

In the present study, 18 Mo strains were isolated from Gansu Province and confirmed as Mo by Giemsa staining, SEM, and 16S rRNA PCR [23, 24]. Since the late twentieth century, molecular typing approaches based on genomic variation have enabled high-resolution discrimination beyond traditional phenotypic methods. With the continuous development of molecular typing techniques, pathogen identification has gradually evolved from PFGE to MLST [12, 13], 16S rRNA gene sequencing [25], and finally to the currently widely used WGS technology. This progression has driven strain characterization from analysis of a limited set of loci toward genome-wide research. In contrast, WGS enables comprehensive characterization of genetic features, evolutionary relationships, and phylogenetic structure [15]. Accordingly, 9 Mo isolates from different farms were subjected to WGS and compared with 168 publicly available Mo genomes derived from sheep-associated and goat-associated lineages retrieved from the NCBI database. Phylogenetic analysis revealed substantial genetic divergence among isolates from different hosts. Notably, the GSWW364 strain identified in this study exhibited close phylogenetic relatedness to the highly virulent strain NJ01 [26] while displaying marked genomic divergence from the reference strain Y98, thereby providing important insights into its genetic background and potential virulence.

Hu sheep represent an economically important dual-purpose breed for meat and lambskin production in China [27]. Recent studies have demonstrated that Mo-associated pneumonia causes considerable economic losses in Hu sheep production systems [6]. Notably, pronounced interindividual variability in susceptibility to Mo infection has been observed within this breed. Therefore, investigating the pathogenicity of clinical isolates and identifying host genetic determinants underlying differential susceptibility are of considerable significance for understanding disease pathogenesis and improving control strategies [28]. In the present study, two intranasal instillations and one intratracheal injection of Mo GSWW364 resulted in successful infection in more than 50% of animals. Infected sheep exhibited characteristic clinical and pathological features of mycoplasmal pneumonia, including pyrexia, coughing, nasal discharge, elevated neutrophil ratios, enlargement of hilar lymph nodes, and pulmonary hepatization, which were in line with the findings of Chen et al. and Zhang et al. [29, 30], thereby confirming the pathogenicity of the GSWW364 strain.

Under identical infection conditions, marked heterogeneity in susceptibility was observed among individuals, reflecting host-specific biological differences. Identification of host genes contributing to this variability is of critical importance for elucidating disease mechanisms and developing targeted interventions. Transcriptomic profiling enables comprehensive characterization of genome-wide gene expression under physiological and pathological conditions, thereby facilitating the identification of key regulatory genes involved in disease processes [18]. Tissue-level transcriptomics captures the integrated responses of multiple cell types, providing a systems-level perspective on disease progression [31], whereas cell-level transcriptomics offers high-resolution insights into cell-specific responses, cellular state transitions, and intercellular interactions. Integration of these complementary approaches enhances the robustness and depth of mechanistic insights [6]. In this study, animals were stratified into susceptible and resistant groups on the basis of infection outcomes, and lung tissues from both groups were subjected to transcriptomic analysis to identify host gene expression differences at the tissue level. Given that SAT II cells represent primary target cells of Mo infection, transcriptomic analysis at the cellular level following Mo exposure provided a refined characterization of host cellular responses. Integrated analysis of tissue- and cell-level datasets identified three candidate genes—*HIF1A*, *SLC2A3,* and *SLC38A1*—as potentially associated with Mo infection.

Accumulating evidence suggests that *HIF1A*, *SLC2A3*, and *SLC38A1* constitute a coordinated regulatory axis in airway inflammation. Hypoxia-inducible factor (HIF)-1α, encoded by *HIF1A*, is a key transcription factor governing cellular adaptation to hypoxia and plays a central role in oxygen homeostasis [32]. It regulates processes including oxygen sensing, glucose transport, amino acid metabolism, and angiogenesis, and exacerbates pulmonary injury via activation of the nuclear factor-kappa B (NF-κB) signaling pathway, thereby promoting neutrophil infiltration and pro-inflammatory cytokine production [33, 34]. *SLC2A3* encodes the glucose transporter 3 (GLUT3) and is a direct transcriptional target of HIF-1α [35, 36]. Additionally, HIF-1α can indirectly upregulate *SLC2A3* via the long noncoding RNA noncoding intergenic co-induced transcript (NICI), further enhancing GLUT3 expression [37]. *SLC2A3* has been identified as an endogenous hypoxia marker in prostate cancer [29, 36] and contributes to airway inflammation in asthma by increasing glucose uptake [38]. *SLC38A1* encodes the amino acid transporter sodium-coupled neutral amino acid transporter 1 (SNAT1), a hypoxia-responsive gene induced by HIF-1α [39], and has been proposed as a potential therapeutic target and early biomarker for acute lung injury [40]. In hypoxic trophoblasts, HIF-1α indirectly upregulates SNAT1 by suppressing miR-373-3p, thereby maintaining cellular proliferation, migration, and amino acid transport functions [41]. This study represents the first investigation of these genes in the context of mycoplasmal pneumonia of sheep. Our preliminary data demonstrated that *HIF1A*, *SLC2A3*, and *SLC38A1* were significantly upregulated in lung tissues of susceptible animals compared with resistant counterparts. At the cellular level, knockdown of each gene resulted in reduced adhesion of Mo to SAT II cells. On the basis of these findings and existing literature, we propose that *HIF1A* may transcriptionally activate *SLC2A3* and *SLC38A1* and act in concert with them to modulate the pathological microenvironment, thereby influencing disease progression. Nevertheless, the precise regulatory interactions between Mo and these host factors warrant further investigation.

In conclusion, this study isolated and characterized 18 Mo strains and performed WGS on nine representative isolates, revealing substantial genetic divergence among strains from different hosts. Notably, GSWW364 exhibited close phylogenetic relatedness to the highly virulent strain NJ01 while differing markedly from the reference strain Y98. Integrated transcriptomic analyses identified *HIF1A*, *SLC2A3*, and *SLC38A1* as candidate host factors potentially involved in susceptibility to Mo infection and disease progression. These findings provide novel insights into host-pathogen interactions and offer a foundation for future studies aimed at elucidating the molecular mechanisms underlying mycoplasmal pneumonia of sheep and improving disease control strategies.

## Supplementary Information

**Additional file 1**. **PCR reaction system.**

**Additional file 2. PCR reaction program.**

**Additional file 3.** **Mo genome sequences.**

**Additional file 4. qPCR reaction system.**

**Additional file 5.** **qPCR reaction program.**

**Additional file 6**.** Collinearity analysis plot.**

## Data Availability

The data of the results in this study are available from the authors upon reasonable request.
